# *Candida albicans* virulence genes SAP1 and SAP6 associated with vaginitis among pregnant women attending a Nigerian tertiary hospital

**DOI:** 10.1016/j.nmni.2022.101029

**Published:** 2022-09-14

**Authors:** O.A. Sobajo, P.A. Okiki, I.P. Ade-Ojo, O. Adelegan, M.I. Adarabioyo

**Affiliations:** 1) Deparment of Biological Sciences, Afe Babalola University Ado-Ekiti, Nigeria; 2) Department of Obstetrics and Gynaecology Ekiti State Teaching Hospital Ado- Ekiti, Nigeria; 3) Statistics Unit, Department of Mathematics and Physical Sciences Afe Babalola University, Ado-Ekiti, Nigeria

**Keywords:** Candida vaginitis, drug resistance, pregnancy, SAP1, SAP6 virulence genes

Dear Editor,

Vaginal candidiasis (VC) in pregnancy is a distressing infection and has emerged as an important cause of neonatal infections. The clinical symptoms and manifestations of VC include cottage cheese-like vaginal discharge, swelling, pruritus, pain, irritation, burning sensation, dyspareunia, and dysuria [1]. The hormonal milieu of the vagina during pregnancy can enhance candidal colonization and serves as risk factor. Progesterone has suppressive effect on the anti-candidal activity of neutrophils, while oestrogen has been found to reduce the ability of vaginal epithelial cells to inhibit the growth of *Candida albicans*. Moreover, a large proportion of women with chronic recurrent candidiasis first experienced the infection during pregnancy. In pregnancy, VC has been related to emotional stress and suppression of immune system [2].

*Candida albicans* is both a commensal and a pathogen that can exhibit yeast, pseudohyphae and hyphae morphology. These morphological transitions promote colonization and invasion at different anatomical sites, which also occur in other *Candida* species. *C. albicans* possesses several virulence factors that are involved in hyphae formation, phenotype switching, cell adhesion and extracellular production of hydrolytic enzymes. Secreted aspartyl proteases (SAPs) are enzymes that are secreted by *Candida* species and are coded for by the SAP gene family (SAP1-SAP10). The SAP superfamily members have been demonstrated as virulence factors in opportunistic pathogens of the genus *Candida*, and SAP1 and SAP6 have been known to be associated with vaginal candidiasis [2,3].

The study was carried out to describe the *Candida* species in vagina of pregnant women, their susceptibility to antifungal agents and the carriage of virulence genes. Pregnant women (390), attending antenatal clinic of Ekiti State University Teaching Hospital, Ado-Ekiti, Nigeria, between 2016 and 2017, were enlisted in the study. Isolation of *Candida* species from high vaginal swab of the subjects was carried out using Brilliance Candida Agar® (Oxoid, England). Antifungal susceptibility testing, on nystatin, fluconazole and variconazole, was performed on the *Candida* isolates by disk diffusion method. *Quick*-DNATM Universal Kit (Zymoresearch, USA) was used for DNA extraction from pure cultures of the *Candida* isolates. The DNA of isolates with green colour on Candida CHROMO agar were subjected to polymerase chain reactions (PCR) using the *C. albicans* specific primers INT1-F:5′-AAGTATTTGGGAGAAGGGAAAGGG-3′ and INT2-R:5′AAAATGGGCATTAAGGAAAAGAGC-3′, to distinguish C. albicans from *C. dublinensis* [4]. PCR amplification of virulence genes SAP 1 and SAP 6 of *Candida albicans* were carried out using the primers: SAP1 (F:5′-TCAATCAATTTACTCTTCCATTTCTAACA-3'; R:5′-CCAGTAGCATTAACAGGAGTTTTAATGACA-3′) and SAP 6 (F:5′-CCCGTTTTGAAATTAAATATGCTGATGG-3'; R:5′-GTCGTAAGGAGTTCTGGTAGC TTC G-3′) as described by Lima et al. [5]. Molecular identification of *Candida* species by PCR analysis and sequencing using the universal primer ITS4/ITS5 (5′-GGAAGTAAAAGTCGTAACAAGG-3’; 5′-TCCTCCGCTTATTGATATGC-3′) as described by El-Naggar *et.al.* [4].

The 390 healthy pregnant women enlisted in this study were 19–46 (30.93 ± 4.60) years old, with median and modal age of 26-32 years. *Candida* species were isolated from HVS of 260 (66.7 %) of the subjects. Based on phenotypical characteristics, the *Candida* species isolated were identified as *C. albicans* (46.8%), *C. dubliniensis* (3.2%), *C. tropicalis* (0.8%), *C. krusei* (29.8%), *C. glabrata* (12.1%) and *C. parapsilosis* (7.3%). The *Candida* isolates showed high level of resistance to the three antifungal agents used; nystatin (50.4 %), fluconazole (79.7 %) and variconazole (81.3 %). Candidal isolation increased with parity, with women after forth delivery recording 75 % compared to 59.6 % of women expecting their first delivery. HIV positive women recorded higher occurrence of *Candida* (75.0 %) than HIV negative individuals (66.4 %). Candidal isolation from HVS of the women was found to be significantly associated with vaginal itching (p < 0.001), vaginal discharge (p < 0.001), previous abortion (p = 0.022) and antibiotics usage (p = 0.044).

Following sequencing and BLAST, the isolates of *C. albicans* were found to belong to different nearest relatives, homology and accession numbers in NBCI data bank. The alignment of the nucleotides of 10 *Candida isolates*, as presented in Fig. 1, showed various sites of insertions, deletions, transversions and translocations. However, 7 out of the 8 *C. albicans* showed strong homology in their nucleotide sequence. The phylogenetic tree shows the ancestral closeness of the 10 isolates of *Candida* species.

**Fig. 1:**
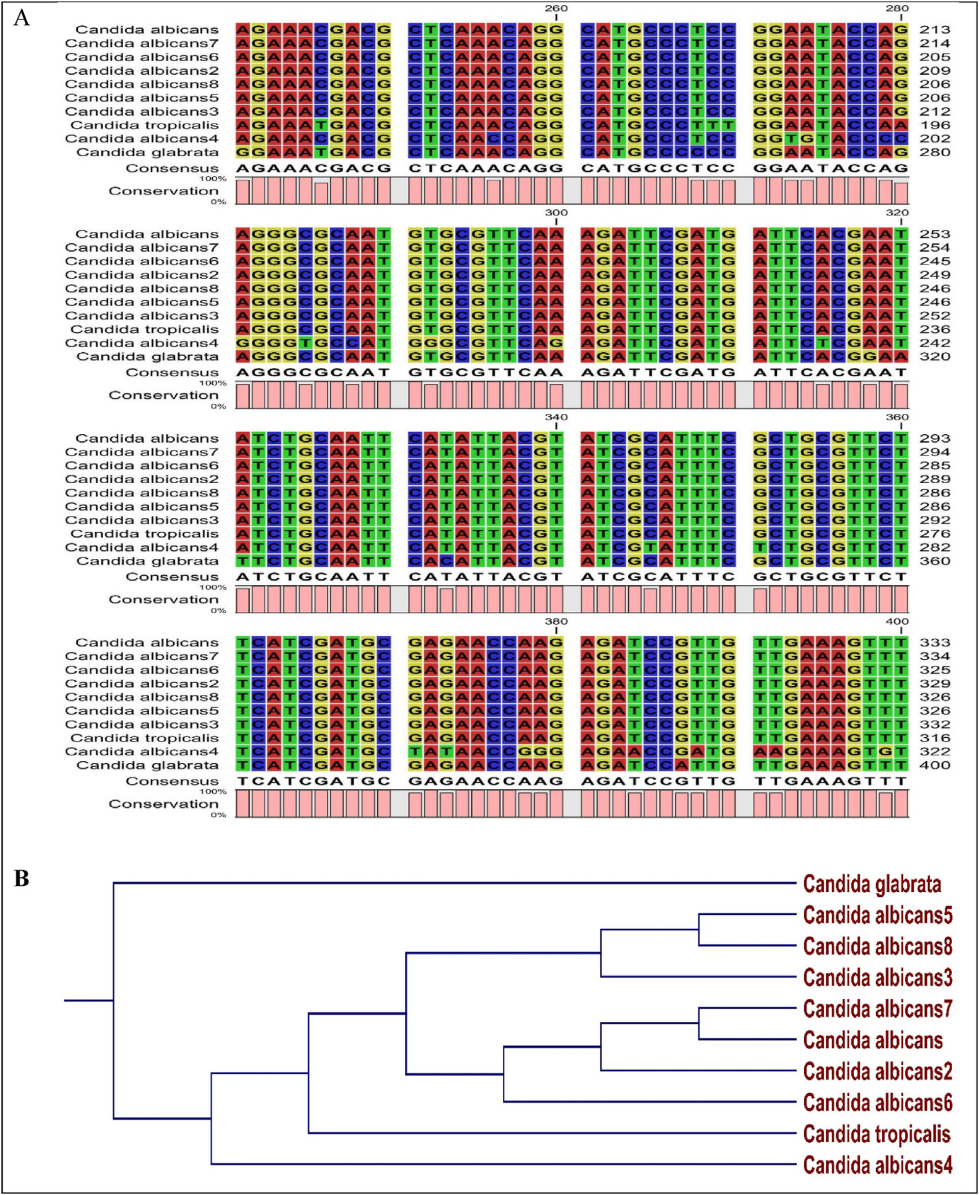
Alignment (A) and phylogenetic relationship (B) among the Candida species isolated from HVS of pregnant women.

The virulence genes SAP1 and SAP6 were detected in 68.75% and 87.50%. respectively among the *C. albicans* isolates and were found to be associated with vaginitis among the subjects. The prevalence of virulence genes SAP1 and SAP6 (68.75 and 87.50 %, respectively), present study is in tandem with the findings in earlier work of Lima et al. [5], who reported incidence of 69.25 and 84.61% respectively of SAP1 and SAP6 genes in *C. albicans* isolated from vulvovaginal infection and colonization in Brazil.

In conclusion, the study showed high prevalence of vaginal candidiasis among apparently healthy pregnant women with drug resistant *Candida* species carrying virulent genes. Perinatal transmission of such drug resistant organisms to new-borns can be of grave consequence.

## Transparency declaration

There is no conflict of interest.
